# Isoliensinine from *Cissampelos pariera* rhizomes exhibits potential gametocytocidal and anti-malarial activities against *Plasmodium falciparum* clinical isolates

**DOI:** 10.1186/s12936-023-04590-7

**Published:** 2023-05-20

**Authors:** Jackson M. Muema, James M. Mutunga, Meshack A. Obonyo, Merid N. Getahun, Ramadhan S. Mwakubambanya, Hoseah M. Akala, Agnes C. Cheruiyot, Redemptah A. Yeda, Dennis W. Juma, Ben Andagalu, Jaree L. Johnson, Amanda L. Roth, Joel L. Bargul

**Affiliations:** 1 Department of Biochemistry, Jomo Kenyatta University of Agriculture and Technology (JKUAT), Nairobi, Kenya; 2 U.S. Army Medical Research Directorate-Africa (USAMRD-A), Centre for Global Health Research (CGHR), Kenya Medical Research Institute (KEMRI), Kisumu, Kenya; 3 Department of Biological Sciences, School of Pure and Applied Sciences, Mount Kenya University, Thika, Kenya; 4 Department of Biochemistry and Molecular Biology, Egerton University, Egerton, Kenya; 5 International Centre of Insect Physiology and Ecology (Icipe), Nairobi, Kenya; 6 Present Address: School of Engineering Design, Technology and Professional Programs, Pennsylvania State University, University Park, PA 16802 USA

**Keywords:** Isoliensinine, Gametocytes, *Plasmodium* transmission-blocking, *Cissampelos pariera*, Malaria control, Natural product

## Abstract

**Background:**

The unmet demand for effective malaria transmission-blocking agents targeting the transmissible stages of *Plasmodium* necessitates intensive discovery efforts. In this study, a bioactive bisbenzylisoquinoline (BBIQ), isoliensinine, from *Cissampelos pariera* (Menispermaceae) rhizomes was identified and characterized for its anti-malarial activity.

**Methods:**

Malaria SYBR Green I fluorescence assay was performed to evaluate the in vitro antimalarial activity against D6, Dd2, and F32-ART5 clones, and immediate ex vivo (IEV) susceptibility for 10 freshly collected *P. falciparum* isolates. To determine the speed- and stage-of-action of isoliensinine, an IC_50_ speed assay and morphological analyses were performed using synchronized Dd2 asexuals. Gametocytocidal activity against two culture-adapted gametocyte-producing clinical isolates was determined using microscopy readouts, with possible molecular targets and their binding affinities deduced in silico.

**Results:**

Isoliensinine displayed a potent in vitro gametocytocidal activity at mean IC_50_^gam^ values ranging between 0.41 and 0.69 µM for *Plasmodium falciparum* clinical isolates. The BBIQ compound also inhibited asexual replication at mean IC_50_^Asexual^ of 2.17 µM, 2.22 µM, and 2.39 µM for D6, Dd2 and F32-ART5 respectively, targeting the late-trophozoite to schizont transition. Further characterization demonstrated a considerable immediate ex vivo potency against human clinical isolates at a geometric mean IC_50_^IEV^ = 1.433 µM (95% CI 0.917–2.242). In silico analyses postulated a probable anti-malarial mechanism of action by high binding affinities for four mitotic division protein kinases; Pfnek1, Pfmap2, Pfclk1, and Pfclk4. Additionally, isoliensinine was predicted to possess an optimal pharmacokinetics profile and drug-likeness properties.

**Conclusion:**

These findings highlight considerable grounds for further exploration of isoliensinine as an amenable scaffold for malaria transmission-blocking chemistry and target validation.

**Supplementary Information:**

The online version contains supplementary material available at 10.1186/s12936-023-04590-7.

## Background

Anticipated therapeutic strategies to block sexual differentiation and maturation of *Plasmodium* gametocytes [1, 2], before uptake by female anopheline mosquitoes would reduce malaria transmissions by magnitude folds [3]. Absence of such effective transmission-blocking interventions has consequently resulted in more than 234 million new infections and 593,000 mortalities reported from sub-Saharan Africa in 2021. *Plasmodium falciparum* gametocytes take about 12–14 days to mature while sequestered in the bone marrow and spleen [4]. In these vascular niches, the parasites display dynamic developmental changes in readiness for a human-to-mosquito phase transition. With such a homing phenomenon, the gametocytes through stages I–V morphological transformations actively remodel host cells reversibly enabling vascular retentions [5–7]. The final maturation of stage IV to V gametocytes is characterized by disassembly of the structural cytoskeleton into rounded tips coinciding with increased cellular deformability induced by cyclic adenosine monophosphate (cAMP) and STEVOR [8, 9]. On phosphorylation of *P. falciparum* STEVOR, the mature stage V gametocytes leave the bone marrow niches and persist for days to weeks in peripheral circulation awaiting mosquito uptake during blood meal acquisition [9]. As previously demonstrated [10–16], *Plasmodium* development is tightly regulated by a robust network of the stage- and sex-specific transcriptional profiles, protein expressions, and physiological metabolic coordination. Despite this vital gametocyte biology and knowledge, drug discovery pace for inhibitors against these transmissible parasites seems quite slow.

Majority of the currently available anti-malarial drugs acting beyond asexual replication fail to completely clear peripherally-circulating mature stage V gametocytes [17], thus enabling transmissions to mosquito vectors [18, 19]. This limitation in addition to current and future anti-malarial resistance trends demonstrate the urgent need for new chemical entities. Over the recent years, various high-throughput screening (HTS) platforms [20–31] have identified diverse chemical scaffolds with high potential for malaria transmission-blocking. Efforts to optimize some of these molecules into lead candidates accompanied by target deconvolutions have yielded KAE609 [32], DDD107498 [33], (+)-SJ557733 [34], ACT-451840 [35], MMV390048 [36] to mention a few, which are currently in early clinical trials. However, pertinent drawbacks related to lack of target and chemical diversity, and high attrition rates are of great concern [37]. Natural products, inclusive of orthogonal anti-malarial herbals [38], have alternatively been pursued for *Plasmodium* transmission-blocking agents. Under these anti-malarial discovery efforts, several natural compounds including: maduramicin [22], parthenin and parthenolide [39], thiostrepton, epoxomicin [40], monensin, salinomycin, nigericin [41], (+)-usnic acid derivatives (BT37 and BT122) [42], naphthyl isoquinoline derivatives [43], azadirachtin A [44], vernodalol [45], 1α,4α-dihydroxybishopsolicepolide [46], p-orlandin [47], cryptolepine [48], lanceolin B [49], dihydronitidine [50], daucovirgolide G [51], and lophirone E [52] have been reported to either kill gametocytes in vitro or prevent their sporogonic development in the mosquito midgut.

**Fig. 1:**
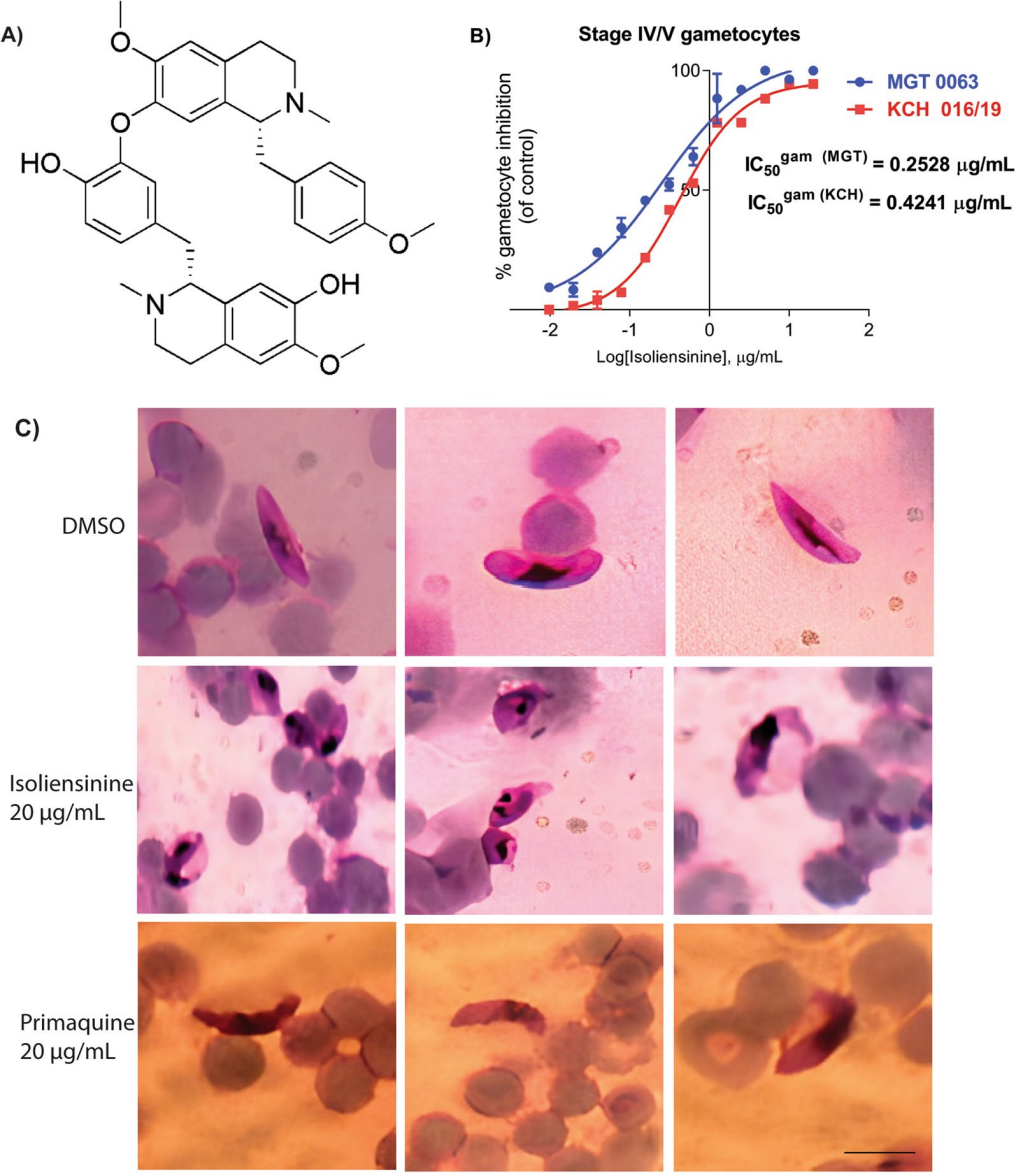
Isoliensinine treatment effects on late-stage IV/V gametocytes from human* Plasmodium falciparum* clinical isolates. **A** Chemical structure of isoliensinine, **B** Isoliensinine reduces stage IV/V gametocytes survival. The dose–response curves of isoliensinine against stage IV/V gametocytes estimated IC_50_^gam^ values to be 0.2528 µg/mL (0.41 ± 0.043 µM), 0.4241 µg/mL (0.69 ± 0.019 µM) for MGT 0063 (Marigat isolate), and KCH 016/19 (Kericho isolate) *Plasmodium* clinical isolates, respectively (n = 3 independent replicates). Error bars indicate standard deviation (s.d). Primaquine served as a positive control in these assays with a mean IC_50_^gam^ 7.46 ± 0.102 µM. **C**: Gametocytes morphological changes on isoliensinine treatment captured from Giemsa-stained slides at 100 × oil immersion objective. Lane 1: 0.2% DMSO-treated gametocytes, Lane 2: isoliensinine-treated gametocytes, and Lane 3: Primaquine treated gametocytes

As part of continued search for new versatile anti-malarial scaffolds, particularly natural products with *Plasmodium* transmission-blocking capability [53], the current screen identified a promising bisbenzylisoquinoline (BBIQ) hit agent. BBIQs, which are typically characterized by tail-to-tail, head-to-head, and head-to-tail structural ether linkage subtypes [54] present multi-ailment drug scaffolds. This particular chemical class preferentially targets both L- and T-type Ca^2+^ signalling pathways in mammalian cells in addition to modulation of membrane efflux channels of ABC transporter and P-glycoprotein (P-gp) families [55, 56]. In *Plasmodium*, mobilization of intracellular Ca^2+^ accompanied by concomitant expression of plant-like effector Ca^2+^-dependent protein kinases, PfCDPK1 – PfCDPK7, initiates timely kinase-specific events; merozoite egress and RBC invasions (CDPK1, CDPK5), asexual growth (CDPK2, CDPK7), activates male exflagellations (CDPK2, CDPK4), ookinetes gliding motility (CDPK3), sporozoites motility and hepatocyte invasion (CDPK6) (reviewed in [57]). In the context of anti-malarial resistance-reversal, previous studies on BBIQs have also demonstrated potential sensitization of quinoline resistant *Plasmodium* parasites through a synergistic action [58, 59]. The underlying CQ sensitization by BBIQs suggests possible modulatory effects on chloroquine (CQ) efflux activity of its transporter PfCRT. Despite exhibiting these interesting pharmacological properties, including potent anti-malarial activities of IC_50_ < 1 µM [59–62], BBIQ-containing compounds have not been pursued further for anti-malarial development to date, perhaps due to their profound structural complexities. Represented among the BBIQs is isoliensinine (Fig. 1A), which was first isolated from lotus seed embryos (*Nelumbo nucifera*, Nelumbonaceae) [63] and later from *Cissampelos mucronata* [64]. The anti-malarial activity profile of isoliensinine is poorly characterized hitherto. Herein, the first detailed anti-malarial characterization of isoliensinine from *Cissampelos pariera* rhizomes (Additional file 1: Fig. S1-S2) has been described and reported as a gametocyte-selective agent with malaria transmission-blocking potential against human *Plasmodium* clinical isolates. The study further describes its relatively slow-acting potent inhibition effect on asexual stages, preferentially acting on mature trophozoite-to-schizont transition step. Possible molecular targets mined and validated through a computational approach highlighted a high affinity for transmembrane transport and mitotic division regulatory proteins. The current findings provide a natural product scaffold with *Plasmodium* transmission-blocking potential amenable to further development into excellent candidates that may be effective additions to the anti-malarial armentarium to treat and prevent malaria transmissions.

## Methods

### Compound acquisition

Anti-malarial drugs; dihydroartemisinin (DHA), mefloquine hydrochloride (MQ), monodesethylamodiaquine (AMQ), artemether (ARM), chloroquine diphosphate (CQ), lumefantrine (LUM), piperaquine tetrahydrate (PPQ), atovaquone (ATQ), artemisinin (ART), artesunate (ARS), quinine (QN), and primaquine (PQ) were sourced from the WorldWide Antimalarial Resistance Network (WWARN). Details on purification and characterization of isoliensinine are included in Additional file 1: Methods S1*.* Unless otherwise stated, all the drug compounds were dissolved in 100% DMSO and reconstituted into desired concentrations for assaying.

### *Plasmodium falciparum* anti-malarial assays

Asexual *P. falciparum* intraerythrocytic parasites; D6, W2, Dd2, and F32-ART5 clones were cultured as previously described [65], with minor modifications. Briefly, parasitized erythrocytes at 0.5% parasitaemia in 4% haematocrit (O^+^ human blood) were cultured at 37ºC with 90% N_2_, 5% CO_2_ and 5% O_2_ in RPMI 1640 medium (Gibco Life Technologies) supplemented with 20% heat-inactivated ABO human serum, 5.94 g/L HEPES (Sigma-Aldrich), 2 g/L glucose, 2 mM l-glutamine, 4 µg/mL hypoxanthine, and 2 g/L NaHCO_3_ (Sigma-Aldrich). To generate highly synchronous cultures, 5% (w/v) D-sorbitol treatments at the ring stage for 10 min at 37 °C were performed. Spent medium was aseptically replaced every two days until attainment of peak parasitaemia level (5–8% rings), during which 100 µL of parasite suspension reconstituted at 1% parasitaemia and 2% haematocrit were dispensed into compound pre-dosed 96-well plates, for 72-h incubation at 37 °C. Final DMSO concentration in all assays did not exceed 0.2% v/v. Replication inhibition activity was analysed in 3 replicates by SYBR Green I-based readouts as previously described [66]. Immediate ex vivo (IEV) susceptibility assay [66] was performed against *P. falciparum* clinical isolates (0–6 h post-phlebotomy collection; at parasitaemia level ≥ 1%) collected from consenting individuals with uncomplicated malaria at Kisumu and Kombewa study site clinics (Approved protocols; KEMRI SSC # 1330 and WRAIR # 1384). Circulating parasites within this endemic West-Kenyan region were previously characterized either mono-/multidrug-resistant or with reduced susceptibility to CQ, sulfadoxine/pyrimethamine (SP), doxycycline (DOX), QN, LUM, and MQ [67–71]. Following an adjustment to 1% parasitaemia, these freshly collected parasites were directly tested without prior culture-adaptation, alongside a panel of standard anti-malarial drugs.

To investigate the speed- and stage-of-action of isoliensinine against asexuals, synchronized Dd2 rings (0–5 h post-invasion (hpi); > 98%), respectively, were examined at 1% parasitaemia against DMSO-treated parasites at different treatment periods within the 48-h intraerythrocytic replication; 5–16, 17–29, and 29–41 hpi [72]. SYBR Green I IC_50_ speed assay was adopted for time specificity of isoliensinine action within the standard 72-h analysis. After each treatment period, stage-specific parasite morphological analyses and imaging of Giemsa-stained thin films were performed.

### Stage IV/V gametocytocidal analysis

Gametocyte induction of culture-adapted human clinical isolates (KCH 016/19 and MGT 0063 from Kericho and Marigat, respectively) was performed according to published method [73]. Giemsa-stained smears were regularly prepared during medium changes on days 5, 8, and 12 post-induction. Percoll-enriched gametocytes (42% stage IV; 58% stage V) at 2% haematocrit were dispensed in 100 µL per well into pre-dosed 96-well plates containing 50 µL of isoliensinine (maximal concentration of 20 µg/mL). 0.2% (*v/v*) DMSO vehicle and PQ were included as negative and positive controls, respectively. The parasites were incubated for 72 h at 37 °C in a humidified atmosphere of 90% N_2_, 5% CO_2_ and 5% O_2_. Thin blood smears were prepared from each well, Giemsa-stained, and percentage gametocyte inhibition determined from light microscopy readouts of 2000–3000 RBCs based on morphology categorization; altered/deformed (dead/dying) versus normal (healthy) [74]. Three independent repeats of experimental analyses were carried out (*n* = 3).

### Computational analyses

Target predictions of isoliensinine were performed on a publicly accessible and curated ChEMBL database (https://www.ebi.ac.uk/chembl/) based on its chemical fingerprints as previously described [75]. Respective target protein sequences were retrieved from UniProt database (https://uniprot.org/) to query for orthology mapping against PlasmoDB (www.plasmodb.org/) using *BLASTp* function under default settings. The resultant *Plasmodium* protein targets were grouped based on the annotated functional roles (molecular function) in reference to published stage-specific transcriptome and proteome data [10]. In order to perform molecular docking for isoliensinine against selected targets, 3D homology models were constructed using the SWISS-MODEL homology-modelling server (https://swissmodel.expasy.org) from protein sequences (.fasta) retrieved from PlasmoDB (Additional file 1: Table S5). The quality of the resultant pdb structural models was assessed using Ramachandran plot parameters. Isoliensinine 2D.sdf file (ZINC42806008) was downloaded as the docking ligand from ZINC15 database. Following energy minimization of isoliensinine by universal force field (uff; 200 steps) under conjugate gradients algorithm and conversion into.pdbqt AutoDock ligand format in OpenBabel tool, docking simulations were performed using AutoDock Vina in PyRx 0.8—Virtual Screening Tool at default X:Y:Z 25 × 25 × 25 Å Vina search space setting. Docking scores for the lowest free binding affinity energies were recorded for each target, ranked, and visualizations of the 2D molecular interactions analysed using Biovia Discovery Studio 2020 Visualizer (v20.1.0.19295; Dassault Systèmes). Isoliensinine ADMET predictions were conducted on SWISSADME (http://www.swissadme.ch).

### Statistical data analysis

Graphpad Prism (GraphPad Prism v.7.0 for windows, San Diego, CA) software was used for data analyses. A non-linear regression model for normalized relative fluorescent units (RFU) readouts was fitted against log_10_-transformed drug concentrations for sigmoidal dose–response plots to estimate IC_50_ values for asexuals. The parameters used were; four parameter logdose with a variable slope: Y = Bottom + (Top–Bottom)/[1 + 10^((LogIC_50_-X) × HillSlope)] fitting. For gametocytocidal IC_50_ determination, average % inhibitions for each treatment doses were plotted against log_10_-transformed doses using non-linear regression analysis [74]. The geometric mean IC_50_ values for continuous immediate ex vivo (IEV) data were analysed with 95% confidence intervals (95% CI) using column statistics. Correlations between IC_50_s values between anti-malarial treatments were computed by Spearman’s nonparametric rank coefficient analysis. Differences between two independent treatment groups were analysed by Student’s *t-test* and a *p* value of less than 0.05 considered statistically significant.

## Results

### Isoliensinine inhibits human *Plasmodium* intracellular growth irrespective of their genetic backgrounds

Initially 13 plant extracts were screened against *P. falciparum* W2 for active anti-malarials. Results obtained by this screening led to the isolation and identification of an active anti-malarial agent in the rhizome extract of *C. pariera* (Menispermaceae), revealed as isoliensinine. Isoliensinine is a potent G1 phase cell cycle inhibitor with anticancer [76], and anti-HIV replication activities [77], alongside a previously reported anti-malarial activity from a congeneric species *C. mucronata* (IC_50_^Asexual^ = 124.3 ng/mL D6; 133.5 ng/mL W2) [64]. As with such aforementioned activity, isoliensinine isolated from *C. pariera* rhizomes was explored for its antiplasmodial activity to pharmacologically characterize its activity profile against both asexual and transmissible sexual stages of *Plasmodium*. From the data presented in Table 1 and Additional file 1: Table S1 – S2, the natural product isoliensinine inhibited intraerythrocytic replication of all the tested parasites in the lower micromolar range (mean IC_50_^Asexual^ = 2.17 µM for D6, 2.22 µM for Dd2, and 2.39 µM for F32-ART5). It is further demonstrated that, similar to the reference anti-malarial drugs; artesunate (ARS), dihydroartemisinin (DHA), atovaquone (ATQ), artemether (ARM), piperaquine (PPQ), and amodiaquine (AMQ), isoliensinine did not exhibit cross-resistance, RI (resistance index) < 10 (Table 1), suggesting a possibly different inhibitory/killing mechanism. Remarkably, isoliensinine treatment displayed a significant fivefold selectivity against late-stage IV/V gametocytes over asexuals (Student’s *t-test*, t = 8.464, *p* = 0.007), with low-micromolar mean IC_50_^gam^ potency values ranging from 0.41 to 0.69 µM (Fig. 1B). This observation appears to suggest better sensitivity and/or uptake of the anti-malarial agent by the less-metabolizing late-stage IV/V gametocytes akin to a recent finding with bichalcone lophirone E from *Lophira lanceolata* (IC_50_^gam^ = 0.14 µM, IC_50_^Asexual^ = 12.23 µM W2, 38.47 µM 3D7) [52]. Morphological distortions induced by isoliensinine consisted of; shrinkage, loss of outer structural outline, and nuclear damage (Fig. 1C).

**Table 1:** Anti-malarial activity of isoliensinine against asexuals of *P. falciparum* laboratory adapted reference clones determined by a malaria SYBR Green I assay

Antimalarial	Mean IC_50_ ± S.D (µM)^a^			Resistance Index (RI)^e^	
	*Pf*D6^b^	*Pf*Dd2^c^	*Pf*F32-ART5^d^	RI_1_	RI_2_
Artesunate	0.003 ± 0.0014	0.002 ± 0.0007	0.004 ± 0.0000	0.667	1.333
Dihydroartemisinin	0.002 ± 0.0000	0.005 ± 0.0000	0.004 ± 0.0006	2.500	2.000
Chloroquine	0.022 ± 0.0007	0.325 ± 0.0321	0.016 ± 0.0005	14.773	0.727
Mefloquine	0.017 ± 0.0049	0.113 ± 0.0014	0.029 ± 0.0027	6.650	1.706
Atovaquone	0.004 ± 0.0003	0.002 ± 0.0007	0.004 ± 0.0004	0.500	1.000
Artemether	0.004 ± 0.0014	0.004 ± 0.0014	0.007 ± 0.0021	1.000	1.75
Piperaquine	0.024 ± 0.0064	0.054 ± 0.0424	0.050 ± 0.0104	2.25	2.083
Amodiaquine	0.008 ± 0.0021	0.008 ± 0.0021	0.007 ± 0.0017	1.000	0.875
Isoliensinine	2.17 ± 0.2775	2.22 ± 0.4097	2.39 ± 0.2001	1.023	1.101

^a^Anti-malarial activity performed for three independent biological replicates

^b^PfD6: Chloroquine sensitive, Mefloquine resistant clone

^c^PfDd2: Chloroquine, Mefloquine, Quinine, Pyrimethamine resistant; Chloroquine sensitive clone

^d^PfF32-ART5: Artemisinin resistant clone

^e^RI_1_ = IC_50_ Dd2/D6; RI_2_ = IC_50_ F32-ART5/D6

S.D—standard deviation

**Fig. 2:**
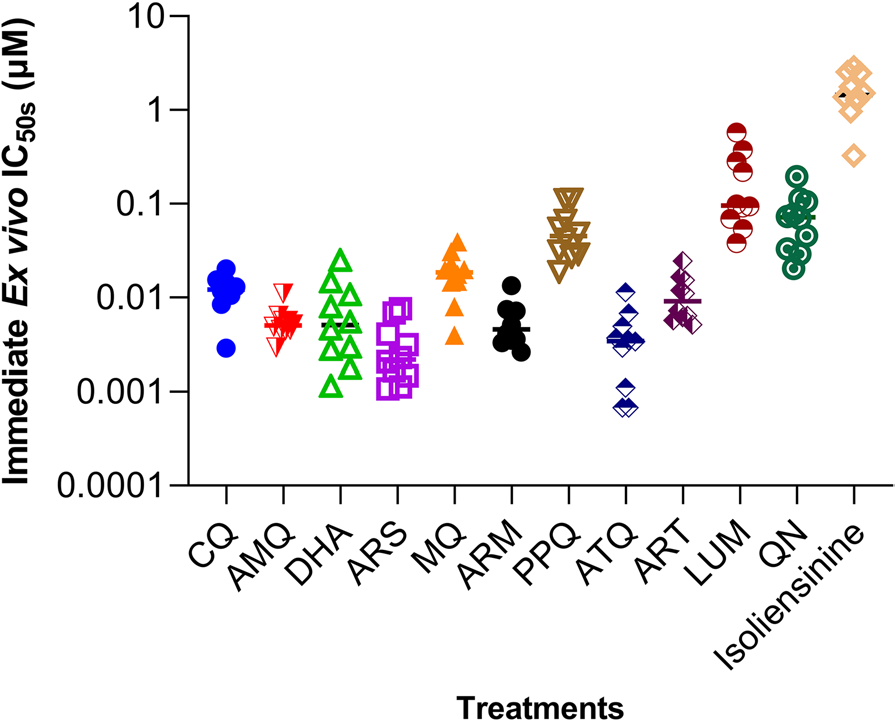
Distribution patterns of immediate ex vivo susceptibilities of *Plasmodium falciparum* clinical isolates to isoliensinine and standard anti-malarial drugs. Geometric mean IC_50s_^IEV^ for the 10 clinical isolates were; CQ = 0.011 µM (95% CI 0.008–0.016), AMQ = 0.005 µM (95% CI 0.004–0.007), DHA = 0.005 µM (95% CI 0.003–0.010), ARS = 0.002 µM (95% CI 0.002–0.004), MQ = 0.016 µM (0.010–0.026), ARM = 0.005 µM (95% CI 0.003–0.007), PPQ = 0.046 µM (95% CI 0.030–0.070), ATQ = 0.003 µM (95% CI 0.001–0.005), ART = 0.010 µM (95% CI 0.007–0.014), LUM = 0.132 µM (95% CI 0.070–0.250), QN = 0.062 µM (95% CI 0.038–0.102), and isoliensinine = 1.433 µM (95% CI 0.917–2.242). Each point represents a single isolate. The horizontal lines between the distribution patterns represent median IC_50s_ for respective treatments

Furthermore, when tested against asexuals of *Plasmodium* derived from human clinical isolates collected from Kisumu region, using immediate ex vivo (IEV) susceptibility assay, isoliensinine consistently maintained its low-micromolar therapeutic efficacy at geometric mean IC_50_^IEV^ = 1.433 µM (95% CI 0.917–2.242, *n* = 10 isolates (Additional file 1: Table S3, Fig. 2). However, no significant inhibitory correlations based on Spearman’s correlation rank coefficient between the paired IC_50s_ values were noted that could imply cross-resistance (Table 2).

**Table 2:** Correlations of immediate ex vivo IC_50s_

Drug pair	Spearman’s rank coefficient (*r*)	*r*^*2*^	*p* value (df)	95% CI
Isoliensinine—CQ	− 0.0061	3.721 × 10^–5^	0.1421 (8)	− 1.0493
Isoliensinine—AMQ	0.1273	0.0162	0.2671 (8)	− 1.1372
Isoliensinine—DHA	− 0.103	0.0106	0.8785 (8)	− 1.2569
Isoliensinine—ARS	− 0.4667	0.2178	0.0647 (8)	− 0.9357
Isoliensinine—MQ	− 0.7455	0.5558	0.4999 (8)	− 1.2135
Isoliensinine—ARM	− 0.4909	0.241	0.5207 (8)	− 1.2178
Isoliensinine—PPQ	0.4424	0.1957	0.3889 (8)	− 1.185
Isoliensinine—ATQ	− 0.4303	0.1852	0.3359 (8)	− 1.167
Isoliensinine—ART	− 0.3697	0.1367	0.2357 (8)	− 1.1203
Isoliensinine—LUM	− 0.0909	0.0083	0.6483 (8)	− 1.2382
Isoliensinine—QN	− 0.1879	0.0353	0.1071 (8)	− 1.0087

### Isoliensinine preferentially targets *Plasmodium* trophozoite-to-schizont transition characterized by a cell division arrest phenotype

**Fig. 3:**
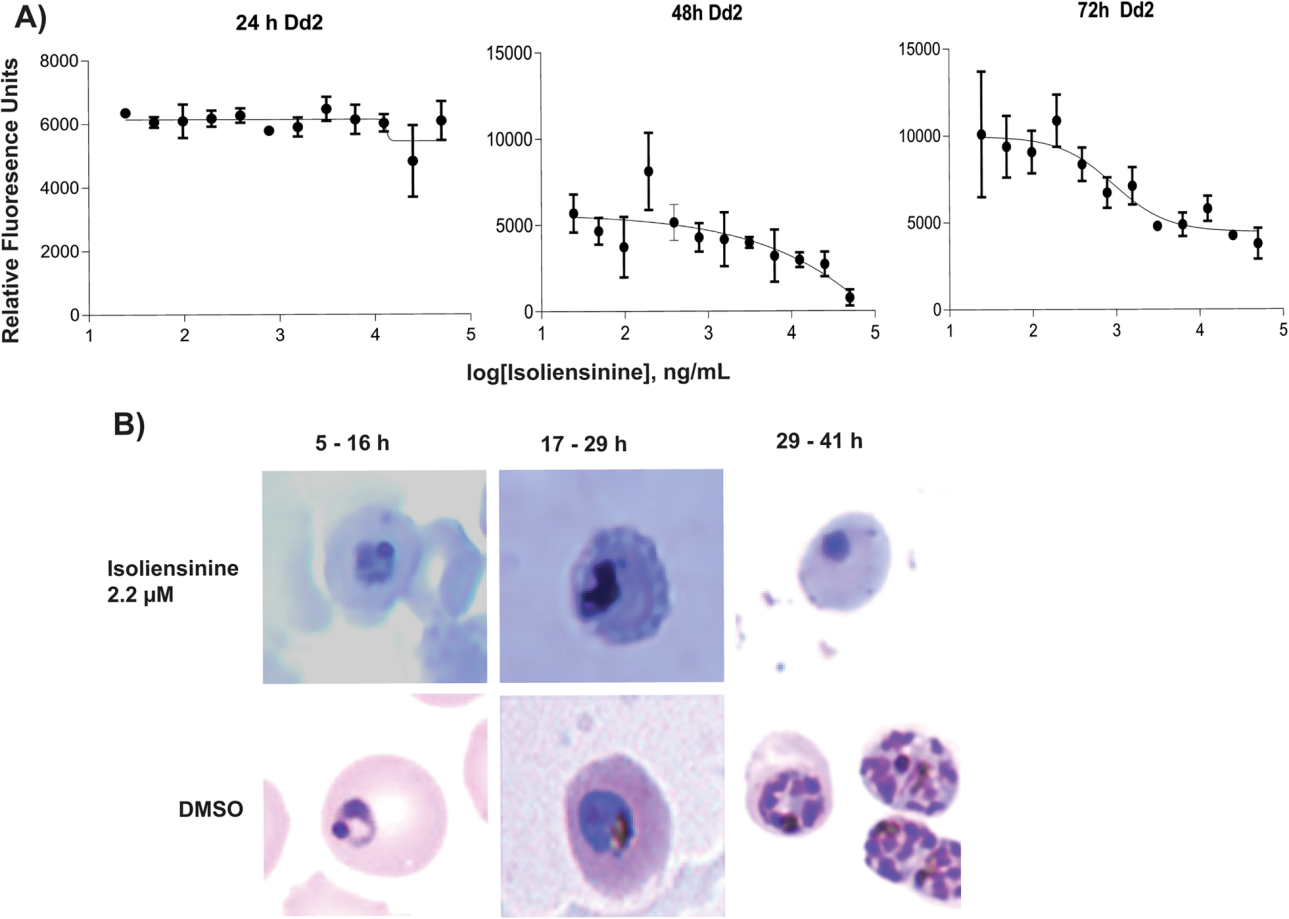
Treatment effects of isoliensinine on *Plasmodium* development. **A** Time course dose–response inhibitory kinetics of isoliensine action against Dd2 in four independent replicates of SYBR Green I IC_50_ speed assay (*n* = 4). Each point represents a mean value of the experimental replicates for the representative periods and error bars indicate standard deviation (s.d). **B** Representative images depicting stage specificity effects exerted against mature trophozoites and schizonts maturation. Smears were made after treatment with isoliensinine at 2.2 µM. Aliquots of synchronized Dd2 parasites at 1% parasitaemia ring stages were treated at 5–16, 17–29, and 29–41 hpi. Morphological examination by light microscopy, under 100 × oil immersion objective, was used to detect the stage specificity of action relative to DMSO-control treatment, as described in the *Methods* section. Three independent experimental replicates were performed (*n* = 3)

After characterization of the anti-malarial profile of isoliensinine against *Plasmodium* parasites, the precise target stage of the ~ 48-h intraerythrocytic cycle was determined. First, a SYBR Green I time specific assay was performed with synchronous Dd2 parasites. During the initial 24-h incubation, the parasites appeared insensitive to isoliensinine treatment portraying a linear curve, but activity appeared between 24 and 48 h and peaked between 48 and 72 h post-incubation with a smooth sigmoid curve (Fig. 3A). In consequence, isoliensinine displayed a relatively slow initial in vitro anti-malarial action akin to late-acting antifolates, sulfadoxine/pyrimethamine, and MMV390048 [36]. To delineate the exact stage-of-inhibition, a highly synchronized Dd2 culture at the ring stage was treated at respective time periods (see *Methods*). The anti-malarial activity was exerted within the first 48-h replication cycle. This precluded the possibility of a delayed death effect exerted by apicoplast-targeting antibiotics such as doxycycline, azithromycin, tetracycline, and clindamycin that occur as a result of disrupted prenylation-dependent intracellular trafficking [78]. *Plasmodium* development failed more pronounceably to progress beyond the late trophozoites indicating critical target(s) at this parasite stage before DNA replication. Morphologically, isoliensinine-treated parasites displayed pyknotic mature trophozoites enclosed by enlarged and blebbed plasma membrane (Fig. 3B), partly portraying effects of targeted ion imbalance associated with ionophores [32, 41]. There were further deleterious effects observed in schizonts, characterized by defective mitotic division exhibiting aggregated nuclear material (Fig. 3B). In tumour cells, isoliensinine has been demonstrated to arrest cell division [76]. Similarly, the treatment of *Plasmodium* Dd2 trophozoites with isoliensinine between 29 and 41 hpi resulted in lack of mature schizont segmenters (Fig. 3B), observed in DMSO-treated parasites. This schizont-specific arrest in nuclear and cell division characterized by non-divided nuclear material suggests impaired mitotic division machinery that otherwise results in 18–24 nuclei of infective merozoites. Such stalled treatment phenotypes resemble those exerted by NITD609 (PfATP4 inhibitor) [32], DDD107498 (protein synthesis—PfeEF2 inhibitor) [33], AN3661 (pre-mRNA processing factor—PfCPSF3 inhibitor) [79], cladosporin (lysyl-tRNA synthetase inhibitor) [80], NED-19 (NAADP inhibition) [81], TCMDC-135051 (mRNA splicing, PfCLK3 inhibitor) [82], aminopyrimidines and oxo-β-carbolines (pre-mRNA splicing, CLKs inhibitors) [83], and tetrathiomolybdate (TTM) (Cu^2+^ homeostasis destabilizer) [84] suggesting similar possible mechanisms.

### Target prediction postulates transmembrane transport and mitotic division axis interactions as a putative mechanism of action for isoliensinine

**Fig. 4:**
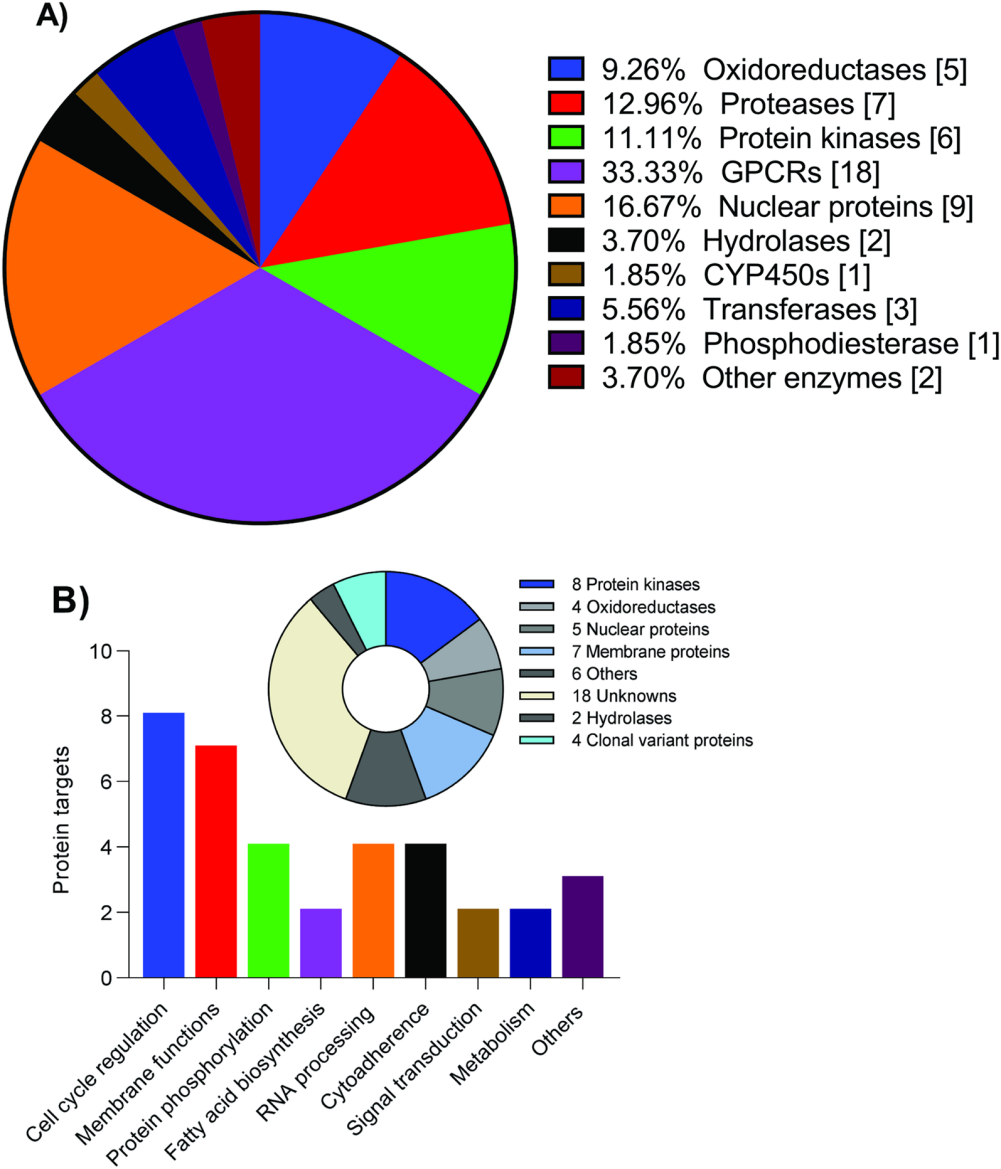
Target analyses formulating mode-of-action hypotheses. **A** Distribution of the 54 predicted target classes affected by isoliensinine. All the “active” labelled mammalian cellular targets predicted in the ChEMBL database at 70–90% confidence for isoliensinine (ID: ChEMBL502370) were recorded and their classes identified. The total number of each class is indicated in the brackets. **B**: Functional classification of the successfully identified *Plasmodium* homologs. *Inset*: Enriched target classes after homology searches in PlasmoDB. Protein sequences of the respective predicted targets retrieved from the UniProt database (using ChEMBL IDs) were used to query PlasmoDB using the *BLASTp* function. The returned orthologs based on identity score were functionally classified utilizing annotations at PlasmoDB and characterization results on published literature

A bioinformatics approach based on historical assays data was deployed to mine for putative protein targets in a publicly accessible ChEMBL database, predicting 54 putative active targets (Additional file 1: Table S4). A majority of these primary candidate targets clustered into G-protein coupled receptors (GPCRs) (18/54; 33.33%), nuclear proteins (9/54; 16.67%), proteases (7/54; 12.96%), protein kinases (6/54; 11.11%), and oxidoreductases (5/54; 9.26%) (Fig. 4A). Functionally annotated *Plasmodium* homologous targets (36) clustered into; protein kinases (8), oxidoreductases (4), nuclear proteins (5), membrane proteins (7), clonal variant proteins (4), hydrolases (2), others (6), and 18 unknowns lacking plasmodial homology (Fig. 4B). The unknown targets were 11 GPCRs, 2 proteases, 1 kinase, 2 hydrolases, and 2 nuclear receptors (Additional file 1: Table S5). When classified based on their plasmodial functions, majority clustered for cell cycle regulation (8), and membrane functions (7), with few enriched for protein phosphorylation (4), RNA processing (4), cytoadherence (4), fatty acid biosynthesis (2), signal transduction (2), metabolism (2), and others (3) (Fig. 4B). Based on these targets, the anti-malarial mechanism of action for isoliensinine was postulated to involve two-step mechanisms at transmembrane trafficking and mitotic division, currently prioritized anti-malarial drug targets under active investigation.

**Fig. 5:**
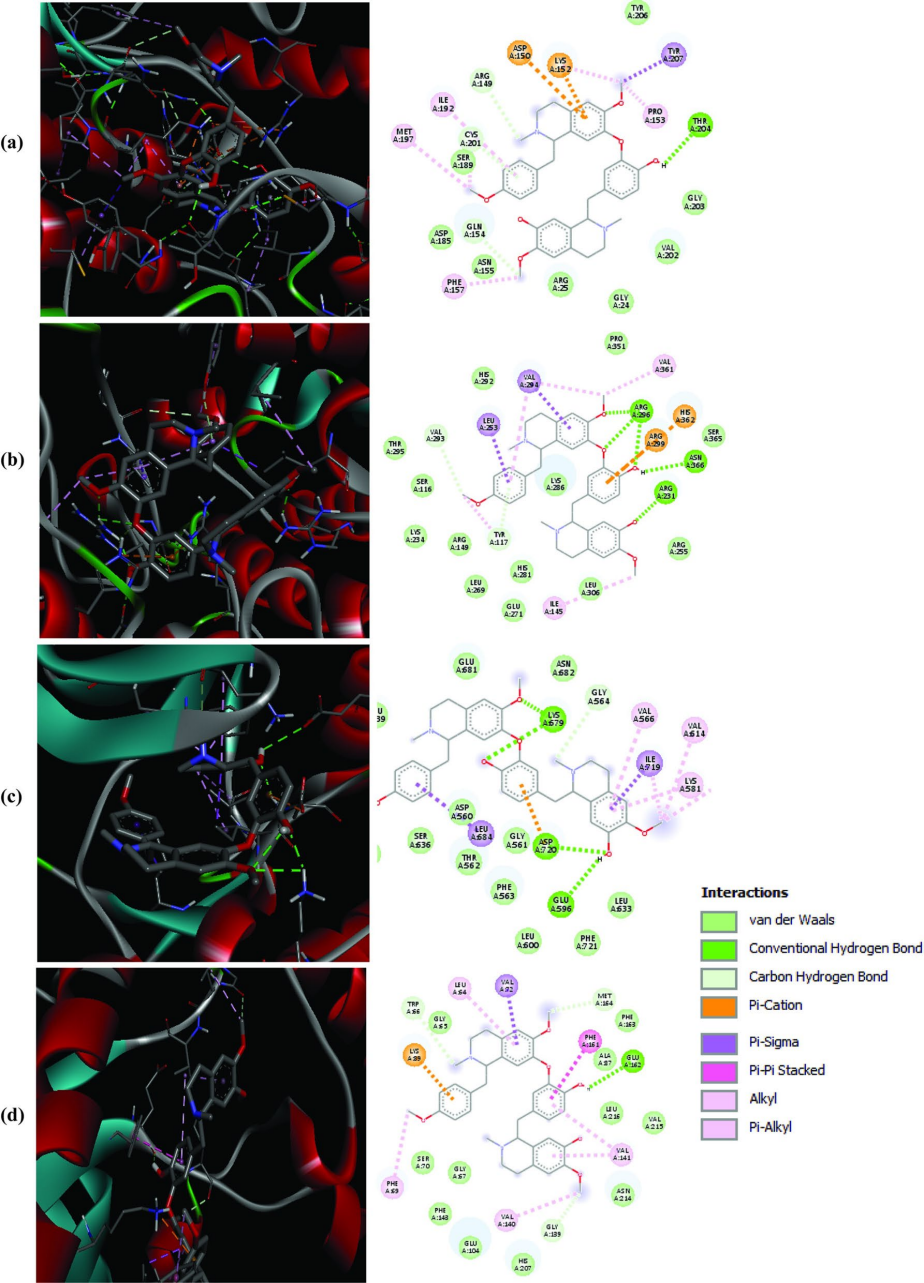
Binding modes of isoliensinine on *Plasmodium* target proteins. **a** PfNek1, **b** PfMap2, **c** PfClk1, and **d** PfClk4. Two dimensional (2D) interaction modes of isoliensinine with the *Plasmodium* protein targets were analysed using Discovery Studio visualizer after molecular docking in PyRx software

To validate the in silico target prediction hypothesis, a reverse molecular docking analysis was performed against 66 *Plasmodium* cell division regulatory proteins and 16 transmembrane transporters, including 4 other targets with similar treatment phenotypes—ATP4, eEF2, CLK3, and CPSF3 (Additional file 1: Table S5). It was established that four prioritized essential protein kinases; 2 Ser/Thr: PfCLK1 (− 10.0 kCal/mol) and PfCLK4 (− 9.8 kCal/mol); 1 Nima: PfNek1 (− 10.8 kCal/mol); and 1 CGMC: PfMap2 (− 10.0 kCal/mol) had high negative free binding energy interactions (< − 9.6 kCalmol^−1^ cut-off) with isoliensinine at their respective binding pockets (Table 3). A single druggable transmembrane lactate/H^+^ symporter, PfFNT, strongly interacted with isoliensinine with a binding energy score of − 9.1 kCal/mol. Isoliensinine demonstrated distinct binding profiles with protein residues of the *Plasmodium* targets utilizing six key bonding interactions; the conventional hydrogen bond, π-cation, π-sigma, π-alkyl, alkyl, and van der Waals forces (Fig. 5).

**Table 3:** Isoliensinine exhibits preferential interaction with four mitotic protein kinases

*Plasmodium* target	PlasmoDB ID	Isoliensinine Binding Affinity (kCal/mol)	Interaction residues
PfNek1	Pf3d7_0525900	− 10.8	Phe157, Gln154, Met197, Cys201, Ile192, Arg149, Asp150, Lys152, Tyr207, Pro153, Thr204
PfClk1	Pf3d7_1445400	− 10.0	Leu684, Asp720, Glu596, Lys679, Gly564, Val566, Ile719, Val614, Lys581, Phe630
PfMap2	Pf3d7_1113900	− 10.0	Val294, Leu253, Val293, Tyr117, Ile145, Arg231, Asn366, Arg299, His362, Arg296, Val361
PfClk4	Pf3d7_0302100	− 9.8	Val140, Phe69, Gly139, Val141, Glu162, Phe161, Met169, Val72, Leu64, Trp66, Lys89

A reverse molecular docking of isoliensinine against 84 selected *Plasmodium* proteins was performed in PyRx—virtual screening tool to determine highly predictive interacting targets based on their free binding energy scores (kCal/mol). Two dimensional (2D) interactions for the best poses were analysed in Discovery Studio visualizer and the results are shown below

### In silico ADME prediction

Finally, the ADME properties of isoliensinine for its drug-likeness, toxicological risks, or pharmacokinetics were profiled using the web-based SWISSADME platform and the results are provided in Additional file 1: Table S6. Isoliensinine was predicted to have an optimal oral bioavailability score, a high intestinal absorption, and non-toxic to five major human metabolic CYP450 isoforms (CYP1A2, CYP2C19, CYP3A4, CYP2C9, and CYP2D6). Additionally, isoliensinine was found impermeant across the blood–brain barrier, but able to inhibit cellular drug efflux mechanisms based on the p-glycoprotein activity. No structural similarities to pan-assay interference compounds (PAINS) were found, however, medicinal chemistry strategies to improve this compound should focus on its solubility in water and reduce the molecular weight to < 500. With such an in silico ADME profile, isoliensinine provides a promising scaffold for drug development devoid of potential liable risks.

## Discussion

The sustained global malaria transmission rates and burden continue to prioritize search for appropriate interventions that could feasibly incapacitate the infectivity of transmissible gametocytes to mosquitoes. Focused on this elusive goal of malaria elimination, and an attempt to expand the existing finite chemical space for this emphasized parasite stage, this study identified a bisbenzylisoquinoline (BBIQ) compound and comprehensively characterized its anti-malarial activity. This BBIQ portrayed a considerable selective gametocytocidal activity. Furthermore, the identified anti-malarial agent inhibited asexual replications by blocking the transition of mature trophozoites into schizonts. The study findings provide the first detailed characterization of the anti-malarial profile of isoliensinine from *C. pariera* rhizomes. Consistent with previous studies that described BBIQs as potential anti-malarials restricted to asexual stages of the parasite [59–62], isoliensinine was also established to inhibit parasite asexuals albeit of slightly lesser potency. Apart from this initial observation, isoliensinine actively inhibited *P. falciparum* parasite isolates from human clinical samples at geometrical mean IC_50_^IEV^ = 1.433 µM. Prior to this study, none of the previously reported BBIQs had been tested against contemporary, clinically-derived parasite isolates. This unprecedented finding has an exciting potential for the management of parasites of highly diverse genetic backgrounds arising from the current selection pressure driven by artemisinin-based combination therapy (ACT) regimens. The current findings clearly demonstrate that isoliensinine possesses impressive gametocytocidal activity in regards to blocking parasite transmissions. Such susceptibility of gametocytes over asexuals to isoliensinine likely points to selective expression of its targets in the former that facilitate better efficacy. Similar selective effects were displayed by dihydroisoquinolone [34], 1α,4α-dihydroxybishopsolicepolide [46], and bichalcones [52]. However, this phenomenon is subject to further interrogation owing to dissimilar stage-specific transcriptomic and proteomic expressions [16].

Turning the attention towards the understanding of the anti-malarial effects displayed by isoliensinine, the inhibitory kinetics and stage specificity of action were determined. Isoliensinine was found to be relatively slow-acting, inhibiting developmental progression of late mature asexual blood stages resulting in collapse of the treated progeny. Unlike the reported findings of a previous study [60], which demonstrated that a related BBIQ cepharanthine from *Stephania rotunda* (Menispermaceae) inhibited ring stages by downregulating Maurer’s clefts, isoliensinine profoundly affected late-stage trophozoite- schizogony transition. This discernible activity discrepancy could indicate structural chemical effects of these compounds. Microscopic examination of the treated parasites suggested that isoliensinine acted during or after the initiation of DNA replication, impairing successful plasmodial nuclear divisions and segregations. Schizont formation corresponding to the M phase is hallmarked by tightly regulated multiple asynchronous mitotic divisions [85], involving various yet unresolved molecular players. While it is generally accepted that BBIQs display a high affinity for divalent cation Ca^2+^-dependent targets, it was however unclear whether isoliensinine followed a similar pathway to exert its anti-malarial effects. But, isoliensinine treatment appeared to compromise important regulatory proteins required for ion homeostasis and mitotic division during schizogony. Of critical emphasis, the exerted phenotype mirrors the anti-malarial effects of various ion channel inhibitors [32, 81, 84]. Although this mechanism could in part be predominant, the observed schizont mitotic failure suggests indirect effector effects by secondary messengers as proposed for a related anticancer BBIQ agent tetrandrine [86]. Free cellular ions like Ca^2+^ drive major cytokinesis events [87], and bidirectional disruption could underscore the observed parasite phenotypes from cytosolic pH imbalance and inhibitory effector signals.

Intrigued by the observed treatment effects and the relative partial exploration of the anti-malarial mechanisms of action of various BBIQs the targets of isoliensinine were computationally mined. Such attempts to elucidate the mechanism of action of isoliensinine based on its chemical fingerprints led us to postulate the involvement of transmembrane transport and mitotic division regulatory proteins, supporting the observed phenotypic treatment effects. The analysis revealed an enrichment of; Pf3D7_0904900 (Cu^2+^ ATPase), Pf3D7_1352100 (Mdr6), Pf3D7_1303500 (Nhe-1), Pf3D7_1235200 (Vp2), and Pf3D7_0830500 highlighting an active interaction with transmembrane solute transporters of both monovalent and divalent cations, as well as amino acids trafficking. Previous studies have demonstrated that solute carrier inhibitors are potent gametocytocidal [22, 30, 41]. As high expression of membrane enriched proteins was reported in late-stage gametocytes [88], and going by the fact that these aforementioned compounds target membrane ion transporters, our data, therefore, support the argument that late-stage gametocytes are likely permeable to ionic homeostasis disruptors. In asexuals, such transmembrane solute transporters maintain cytosolic ion homeostasis, and attractive drug targets for various anti-malarials [89]. Some of these transmembrane ion transporters are refractory to conditional gene deletions hence indispensable for parasite schizogonic replications. Guided by the strong morphological distortions, it was therefore unlikely to exclude a possible similar mechanism involving transmembrane transport from isoliensinine anti-malarial action.

Furthermore, discernible effects on treated schizonts that failed to divide their nuclear material were noted. Target enrichment of the predicted *Plasmodium* proteins showed the highest number of previously characterized cell cycle regulators that could underscore the observed developmental arrest. For example, chromatin modifiers; Pf3D7_1211600 (LSD1), Pf3D7_0809900 (JmjC1), and Pf3D7_1212900 (BDP2) peak their expressions during schizonts [90], and reasonable that the corresponding effects of isoliensinine at this stage were more pronounced. Drugs targeting epigenetics [91], exert promising anti-asexual and gametocytocidal activities. Among other druggable targets identified through the predictions required for successful schizont development are the Ca^2+^-dependent cysteine protease calpain (Pf3D7_1362400), PI4K (Pf3D7_0509800) [92], dfhr-TS (Pf3D7_0417200), CDPK7 (Pf3D7_1123100), PKB (Pf3D7_1246900), and casein kinase 2 (Pf3D7_1108400) [93]. However, from the molecular docking analyses, a remarkable affinity for four essential mitotic division protein kinases; PfNek1, PfCLK1, PfCLK4, and PfMap2 was noted. Orthologs for these protein kinases stabilize kinetochore-microtubule attachment of centrosomes to regulate protozoan mitotic events and cell cycle progression [94, 95], providing opportunities for drug targeting. In *Plasmodium*, the cyclin-dependent kinase-like kinases (CLKs) phosphorylate serine/arginine-rich pre-mRNA splicing factor substrates. Their chemical knockouts have been reported to inhibit trophozoite-schizont transition, impair gametocyte development, and reduced male gamete exflagellations and mosquito infection prevalence to 50% [82, 83]. The *Plasmodium* never-in-mitosis gene A (PfNek1) regulates cell cycle at trophozoite/schizont transition and male gametocytes [96], while PfMap2 is expressed during asexual, ookinetes, and essential for male gametogenesis [97]. Reasonably, it is not surprising thereof the potential interaction of isoliensinine with these *Plasmodium* protein kinases could have led to similar treatment results.

As the in silico approaches to ADME properties for a new bioactive compound could formulate strategies towards its structural optimization, the analyses demonstrated an acceptable pharmacokinetics profile for isoliensinine. Further prioritization studies aimed at improving its drug-likeness were identified to incline towards solubility in water and reduction of molecular weight to the acceptable < 500 limit.

The prime validation strategy of malaria transmission-blocking activity for any drug candidate is predominantly through a mosquito standard membrane feeding assay (SMFA). However, it is hereby considered that interrupting schizonts formation and maturation could reduce *Plasmodium* gametocytaemia. Additionally, the selective killing of late-stage IV/V gametocytes by isoliensinine prospects a remarkable pathway towards reducing infective parasites and future SMFA explorations of this anti-malarial agent. SMFAs will further inform on the potential dissemination mechanisms for roll-out of technologies deploying isoliensinine to target vectors or at the human host interface. In summary, the findings demonstrated that isoliensinine, a BBIQ isolated from *C. pariera* rhizomes, is an anti-malarial agent that is selectively gametocytocidal. This study recommends the limitation of microscopy readout for estimation of the gametocytocidal activity be resolved and validated by better fluorescence assays. Still, the provided evidence supports further characterization and development efforts of this promising malaria transmission-blocking lead scaffold with optimal ADME profile.

## Supplementary Information

**Additional file 1: Methods S1; Table S1:** Anti-malarial screening of plant extracts using SYBR Green I assay; **Table S2:** Anti-malarial activity readouts of *C. pariera* solvent fractions; **Table S3:** Immediate ex vivo susceptibilities of *Plasmodium* clinical isolates to isoliensinine relative to standard anti-malarial drugs in µM; **Table S4:** Predicted isoliensinineprotein targets; **Table S5:** Molecular docking of *Plasmodium* targets to isoliensinine; **Table S6:** ADME prediction profile enlisting of isoliensinine from SWISSADME platform. **Fig. S1:** *Cissampelos pariera* in its natural ecosystem and the root rhizomes; **Fig. S2:** LC–MS/MS fragmentation of isoliensinine.

## Data Availability

All data generated or analysed during this study are included in this article and its additional information files.
