# Supplementary material for: Isoliensinine from Cissampelos pariera rhizomes exhibits potential gametocytocidal and anti-malarial activities against Plasmodium falciparum clinical isolates
Source: Malar J. 2023 May 20;22:161. doi: 10.1186/s12936-023-04590-7 (PMC10199507; doi:10.1186/s12936-023-04590-7)
Supplement: Supplementary file 1 — Additional file 1: Methods S1; Table S1: Anti-malarial screening of plant extracts using SYBR Green I assay; Table S2: Anti-malarial activity readouts of C. pariera solvent fractions; Table S3: Immediate ex vivo susceptibilities of Plasmodium clinical isolates to isoliensinine relative to standard anti-malarial drugs in µM; Table S4: Predicted isoliensinineprotein targets; Table S5: Molecular docking of Plasmodium targets to isoliensinine; Table S6: ADME prediction profile enlisting of isoliensinine from SWISSADME platform. Fig. S1: Cissampelos pariera in its natural ecosystem and the root rhizomes; Fig. S2: LC–MS/MS fragmentation of isoliensinine. [file 12936_2023_4590_MOESM1_ESM.doc]

**Supplementary information**

**Isoliensinine from *Cissampelos* *pariera* rhizomes exhibits potential gametocytocidal and anti-malarial activities against *Plasmodium falciparum* clinical isolates**

Jackson M. Muema^1,2*^, James M. Mutunga^2,3,6^, Meshack A. Obonyo^4^, Merid N. Getahun^5^, Ramadhan S. Mwakubambanya^4^, Hoseah M. Akala^2^, Agnes C. Cheruiyot^2^, Redemptah A. Yeda^2^, Dennis W. Juma^2^, Ben Andagalu^2^, Jaree L. Johnson^2^, Amanda L. Roth^2^, & Joel L. Bargul^1,5*^

**Affiliations;**

^1^Department of Biochemistry, Jomo Kenyatta University of Agriculture and Technology (JKUAT), Nairobi, Kenya

^2^U.S. Army Medical Research Directorate-Africa (USAMRD-A), Centre for Global Health Research (CGHR), Kenya Medical Research Institute (KEMRI), Kisumu, Kenya

^3^Department of Biological Sciences, School of Pure and Applied Sciences, Mount Kenya University, Thika, Kenya

^4^Department of Biochemistry and Molecular Biology, Egerton University, Egerton, Kenya

^5^International Centre of Insect Physiology and Ecology (*icipe*), Nairobi, Kenya

^6^Present address: School of Engineering Design, Technology and Professional Programs, Pennsylvania State University, University Park, PA, 16802, USA

To whom correspondence should be addressed;

[Jackson_mbithi@yahoo.com](mailto:Jackson_mbithi@yahoo.com) and [jbargul@jkuat.ac.ke](mailto:jbargul@jkuat.ac.ke) or [jlbargul@gmail.com](mailto:jlbargul@gmail.com)

***Methods S1***

**Isolation and characterization of Isoliensinine**

Initial activity-based screening of various plant-based extracts against *Plasmodium* W2 strain revealed promising antimalarial activity (IC_50_ < 2.5 µg/mL) with *Cissampelos* *pariera* (Menispermaceae; S1 Fig) root extract (S1 Table). The plant materials (rhizomes; S1 Fig) were sampled in August 2018 from Kiritiri, Mbeere South Constituency in Embu County (GPS coordinates: 00˚42'54.6''S, 037˚38'28.1''E, 1561 m a.s.l) and authentically identified by Mr. Patrick C.B. Mutiso of the University of Nairobi Herbarium, School of Biological Sciences (voucher specimen reference number: JMM2018/10). No specific authorization permits were sought for the sample collections since the plant is not classified as endangered or protected species. 40.0 g of the CH_2_Cl_2_/MeOH (1:1, v/v) *C.* *pariera* root extract were adsorbed in silica gel (Kiesegel 60 M, [0.004-0.063 mm mesh size], Macherey-Nagel GmbH & Co.KG, Düren, Germany) and chromatographed over silica gel-packed column chromatography (CC). An increasing gradient elution of *n*-hexane/EtOAc (1:0, 9:1, 7:3, 1:1, 3:7, 1:9, 0:1) followed by EtOAc-MeOH (1:1, 1:9, and 0:1) yielded 40 100-mL fractions. The fractions were pooled into 8 major fractions (Fr. CP01-CP08) based on thin layer chromatography (TLC) profiling. Antimalarial active fraction CP08 (3.63 g) was further re-chromatographed over silica gel CC with MeOH/H_2_O (13:7, 7:13, 0:1) to yield a yellowish - brown amorphous powder (2.74 g; 6.85% yield).

1 mg of the isolate was reconstituted in HPLC-grade Chromasolv® MeOH/H_2_O (1:1, v/v), vigorously vortexed, and centrifuged for 5 min at 14000 rpm (25˚C). 1-mL of the supernatant was subsequently transferred into an autosampler HPLC vial and 0.005 mL automatically injected for analysis. Peak discriminatory analysis was performed using diluent solvents (MeOH/H_2_O). LC-ESI-MS/MS analysis was performed on Agilent’s 1290 Infinity series UHPLC fitted with a rapid resolution reverse-phase C_18_ column (1.8 µm, 2.1 × 150 mm i.d; Agilent Technologies) interfaced with a 6490 model Triple Quad MS system. The MS system was equipped with iFunnel JetStream electron source operating in the positive ionization mode using dynamic multi-reaction monitoring (MRM) software features. During the chromatographic analyses, the column temperature was maintained at 45˚C. An elution gradient profile with a binary solvent mobile phase of H_2_O (A) and MeOH (B) maintained at a flow rate of 0.4 mL/min was programmed as follows: 95%-5% (0-3.5 min), 50%-50% (3.5-17 min), 0%-100% (17-20 min), and 95%-5% (20.1-25 min). Analysis was performed under ESI settings; capillary voltage of 3.4 kV, gas flow rate of 11.0 L/min, gas temperature of 200˚C, sheath gas flow rate of 8.0 L/min, nebulizer pressure of 30 psi, and sheath gas temperature of 375˚C. Nitrogen was used as both the nebulizer and collision gas. Full MS/MS spectra scans of m/z 10-1000 Da range were acquired and analysed by Agilent MassHunter Qualitative analysis software (version B.06.00).

The compound identity was inferred from its retention time, mass-to-charge ratio (m/z), mass fragmentation profile (S2 Fig), and by comparison with published literature (1).

**References**

1. Bala, M. *et al.* 2019. Bioactive isoquinoline alkaloids from *Cissampelos pariera*. *Nat. Prod. Res.* **33,** 622-627

**Fig. S1: *Cissampelos pariera* in natural ecosystem and its root rhizomes**


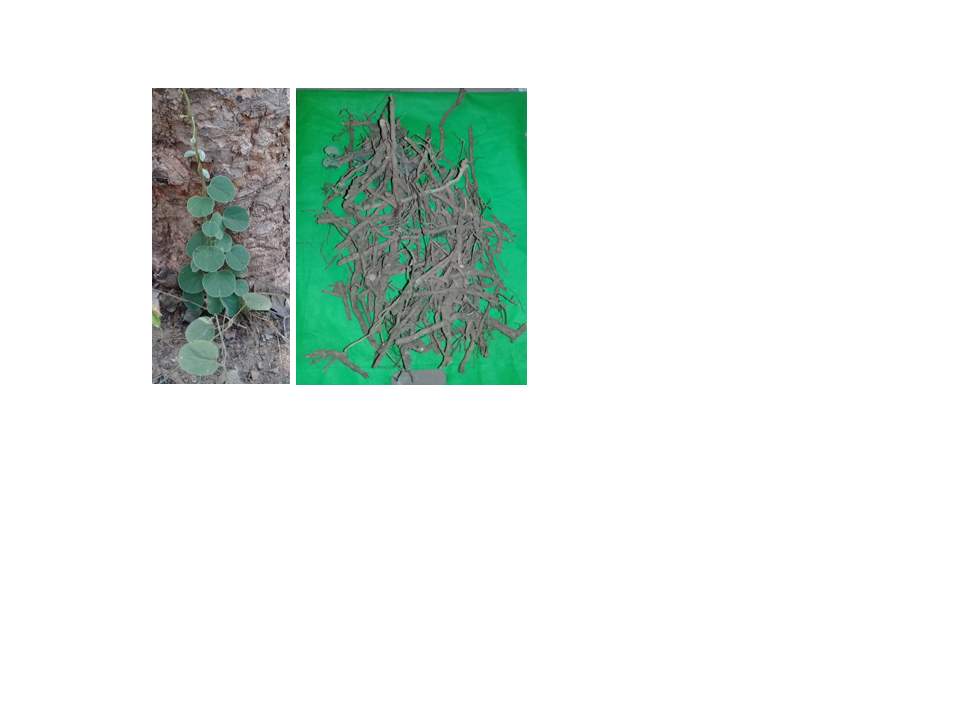


**Fig. S2: LC-MS/MS fragmentation of isoliensinine**


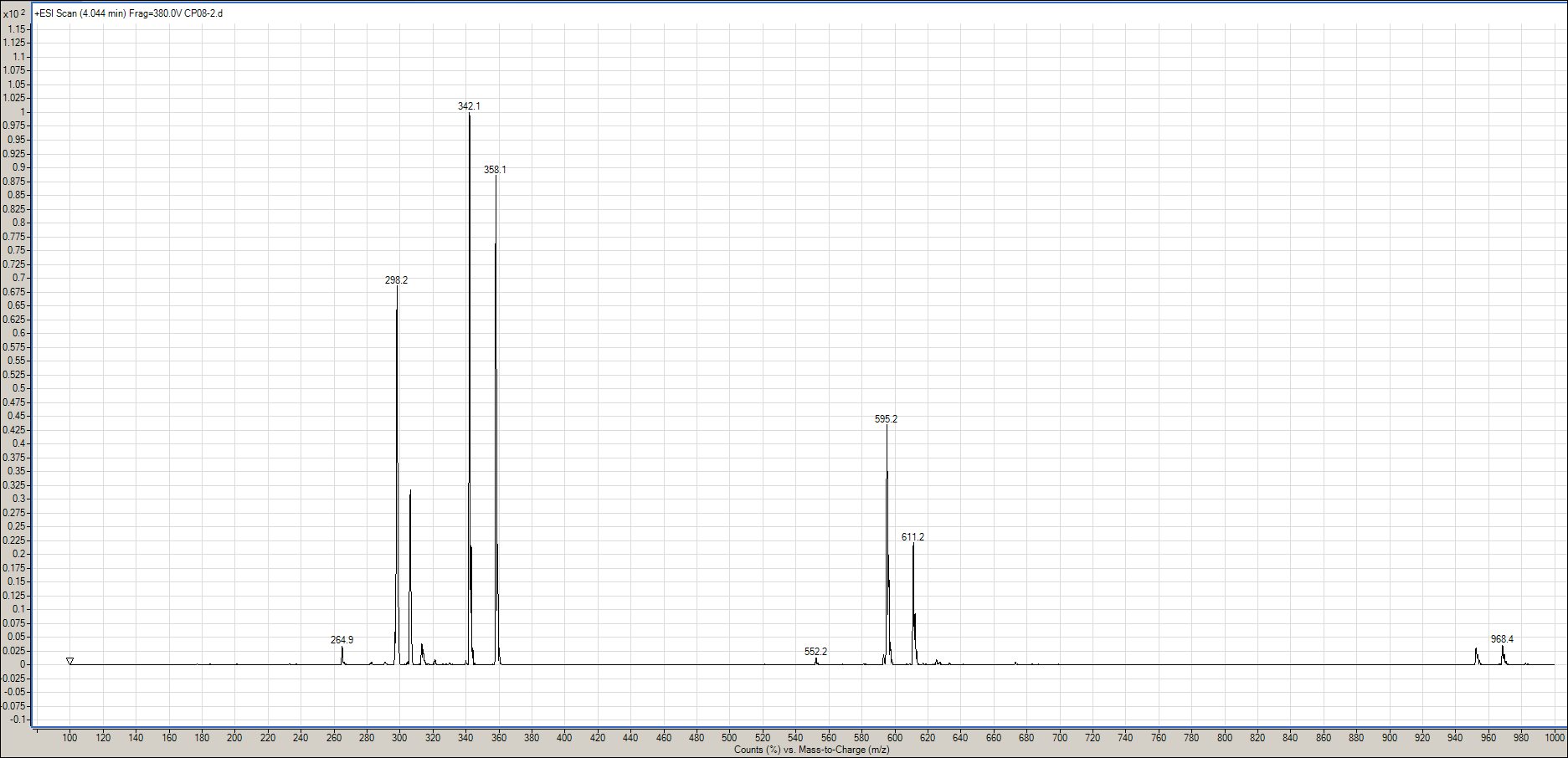


**Table S1**: Antimalarial screening of plant extracts using SYBR Green I assay.

| **Plant species** | **Family** | **Accession number** | **Part used** | **Extract** | **W2 IC_50_ (µg/mL)^a^** |
| --- | --- | --- | --- | --- | --- |
| *Cissampelos pariera* | Menispermaceae | JMM2018/10 | Rhizomes | CH_2_Cl_2_/MeOH (1:1) | 2.09 ± 0.21 |
| *Vitex payos* | Verbenaceae | JMM2018/04 | Root bark | Acetone | 15.50 ± 12.95 |
| *Mangifera indica* | Anacardiaceae | JMM2018/03 | Stem bark | 95% Ethanol | >1000 |
| *Camelia sinensis* | Theaceae | JMM2018/13 | Leaves | Acetone/H_2_O (7:3) | >1000 |
| *Vitex schiliebenii* | Verbenaceae | JMM2018/05 | Leaves | Acetone | >500 |
| *Prosopis juliflora* | Fabaceae | JMM2018/08 | Leaves | CH_2_Cl_2_/MeOH (1:1) | 1.02 ± 0.02 |
| *Zanthoxylum chalybeum* | Rutaceae | JMM2018/06 | Root bark | CH_2_Cl_2_/MeOH (1:1) | 164.83 ± 216.68 |
| *Chrysathemum cinerarifolium* | Compositae | JMM2018/02 | Flowers | n-Hexane | 310.29 ± 14.47 |
| *Terminalia brownii* | Combretaceae | JMM2018/09 | Stem bark | CH_2_Cl_2_/MeOH (1:1) | 28.88 ± 19.86 |
| *Persea* *americana* | Lauraceae | JMM2018/07 | Seeds | Acetone/H_2_O (7:3) | >500 |
| *Agerantum conyzoides* | Asteraceae | JMM2018/12 | Whole aerial shoot | CH_2_Cl_2_/MeOH (1:1) | 36.01 ± 23.51 |
| *Schinus terebinthifolius* | Anacardiaceae | JMM2018/01 | Leaves | CH_2_Cl_2_/MeOH (1:1) | 79.43 ± 3.71 |
| *Murraya koenigii* | Rutacae | JMM2018/11 | Leaves | CH_2_Cl_2_/MeOH (1:1) | >1000 |

**^a^**Mean IC_50_ derived from 2 independent experimental screenings.

Collection sites for the above study materials of the other plants (apart from *C.* *pareira*) were as follows; *Vitex payos, Mangifera indica, Zanthoxylum chalybeum,* and *Terminalia brownii* were collected from Mathemba village (Makueni County) geo-referenced at; 01˚53'32.1''S, 037˚45'33.0''E, 1086 m asl; 01˚53'27.9''S, 037˚45'06.7''E, 1133 m asl; 01˚53'22.1''S, 037˚45'11.9''E, 1109 m asl, and 01˚53'22.7''S, 037˚45'13.9''E, 1111 m asl, respectively. *Camellia* *sinensis* leaves were purchased from a Limuru Archdiocese tea farm (GPS coordinates: 01˚07'10''S, 036˚39'37''E, 2225 m asl). *Vitex* *schiliebenii* leaves were collected from Gede town in Kilifi County, near Arabuko-Sokoke rain forest off the Kenyan coast (GPS coordinates: 03˚18'08.3''S, 039˚59'51.8''E, 34 m asl). Leaves of *Prosopis* *juliflora* were collected from Marigat town in Baringo County (GPS coordinates: 00˚28'25.6''N, 035˚58'49.5''E, 1042 m asl). We purchased *Chrysathemum* *cinerarifolium* (pyrethrum) flowers from a local farmer at Kinungi East near Naivasha town (GPS coordinates: 00˚45'30.9''S, 036˚30'41.5''E, 2318 m asl). The seeds of *Persea* *americana* (avocado) were collected from Kandara in Murang’a County (GPS coordinates: 00˚57'46.0''S, 037˚05'26.4''E,1536 m asl).We collected *Agerantum* *conyzoides* from JKUAT main campus (GPS coordinates: 01˚05'56.3''S, 037˚00'51.3''E, 1525 m asl), leaves of Brazilian pepper (*Schinus* *terebinthifolius*) from *icipe* Duduville campus in Nairobi (GPS coordinates: 01˚13'17.8''S, 036˚53'47.2''E, 1603 m asl ) and *Murraya* *koenigii* from Kibwezi in Makueni County (GPS coordinates: 02˚23'13''S, 038˚00'03''E, 899 m asl).

**Table S2: Antimalarial activity readouts of *C. pariera*** solvent fractions

| **Fraction code** | **KOM 145^a^** | **KOM 148^a^** | **KDH 0163^a^** | ***Plasmodium* D6** |
| --- | --- | --- | --- | --- |
| **CP01** | 72.722 | Inactive | Inactive | ND |
| **CP02** | Inactive | Inactive | Inactive | ND |
| **CP03** | Inactive | 18.451 | Inactive | ND |
| **CP04** | Inactive | 24.709 | Inactive | ND |
| **CP05** | 32.796 | Inactive | Inactive | ND |
| **CP06** | Inactive | 21.615 | 31.405 | ND |
| **CP07** | 24.552 | 15.840 | 18.113 | ND |
| **CP08** | 1.078 | 0.2 | 1.517 | 1.329 |

**^a^**IC_50_ determined for *Plasmodium* clinical isolates assayed by Immediate *Ex vivo* SYBR readout in µg/mL. ND – Not determined. Inactive – IC_50_ > 100 µg/mL

**Table S3: *Immediate* *Ex* *vivo* susceptibilities of *Plasmodium* clinical isolates to isoliensinine relative to standard antimalar**ial drugs in µM

| ***P. falciparum* isolates** | **CQ** | **AMQ** | **DHA** | **ARS** | **MQ** | **ARM** | **PPQ** | **ATQ** | **ART** | **LUM** | **QN** | **Isoliensinine** |
| --- | --- | --- | --- | --- | --- | --- | --- | --- | --- | --- | --- | --- |
| **Field isolate 1** | 0.012646 | 0.006399 | 0.007962 | 0.002083 | 0.019949 | 0.005084 | 0.027208 | 0.00292 | 0.015372 | 0.282662 | 0.113094 | 0.963 |
| **Field isolate 2** | 0.014437 | 0.004904 | 0.004639 | 0.003137 | 0.0309 | 0.004099 | 0.029188 | 0.00371 | 0.005122 | 0.375307 | 0.045836 | 1.36 |
| **Field isolate 3** | 0.011665 | 0.00451 | 0.001146 | 0.001664 | 0.022799 | 0.0036 | 0.110663 | 0.003386 | 0.024585 | 0.094063 | 0.073393 | 1.512 |
| **Field isolate 4** | 0.002892 | 0.004485 | 0.002967 | 0.001075 | 0.014676 | 0.003509 | 0.066536 | 0.011411 | 0.011012 | 0.053885 | 0.105604 | 1.385 |
| **Field isolate 5** | 0.010582 | 0.01106 | 0.001779 | 0.001472 | 0.007948 | 0.002614 | 0.048422 | 0.00675 | 0.006145 | 0.575912 | 0.194316 | 2.858 |
| **Field isolate 6** | 0.015429 | 0.005671 | 0.0055 | 0.001129 | 0.017663 | 0.007192 | 0.111709 | 0.003506 | 0.016495 | 0.038268 | 0.03292 | 2.558 |
| **Field isolate 7** | 0.012999 | 0.00453 | 0.002799 | 0.006873 | 0.014869 | 0.007467 | 0.03141 | 0.00495 | 0.005699 | 0.069087 | 0.070063 | 0.327 |
| **Field isolate 8** | 0.011293 | 0.00566 | 0.014704 | 0.007572 | 0.003981 | 0.013336 | 0.054678 | 0.001107 | 0.006393 | 0.098298 | 0.020384 | 1.274 |
| **Field isolate 9** | 0.008447 | 0.005182 | 0.010782 | 0.004089 | 0.039359 | 0.003298 | 0.01888 | 0.000679 | 0.011912 | 0.219134 | 0.029046 | 1.765 |
| **Field isolate 10** | 0.020198 | 0.002956 | 0.024959 | 0.002301 | 0.01964 | 0.00676 | 0.042017 | 0.000677 | 0.007247 | 0.091681 | 0.077369 | 2.484 |
| **Geometric mean IC50** | 0.010968 | 0.005241 | 0.005169 | 0.002499 | 0.016301 | 0.005059 | 0.046072 | 0.002752 | 0.009621 | 0.132089 | 0.061892 | 1.433422776 |
| **25th percentile** | 0.010759 | 0.004515 | 0.002841 | 0.00152 | 0.014724 | 0.003532 | 0.029743 | 0.00156 | 0.006207 | 0.074735 | 0.036149 | 1.2955 |
| **75% percentile** | 0.014077 | 0.005668 | 0.010077 | 0.003851 | 0.022086 | 0.007084 | 0.063571 | 0.00464 | 0.014507 | 0.26678 | 0.098545 | 2.30425 |
| **Median IC50** | 0.012155 | 0.005043 | 0.005069 | 0.002192 | 0.018651 | 0.004592 | 0.045219 | 0.003446 | 0.009129 | 0.096181 | 0.071728 | 1.4485 |

**Table S4: Predicted isoliensinine (CHEMBL**502370) protein targets

| **ChEMBL ID^a^** | **Mammalian target name** | **Target Class** |  | ***Plasmodium* homologue ID^b^** | **Cellular function in *Plasmodium*** |
| --- | --- | --- | --- | --- | --- |
| CHEMBL4358 | Arachidonate 15-lipoxygenase | Oxidoreductase |  | Pf3D7_0419600 | Intracellular transport |
| CHEMBL4244 | Legumain | Cysteine protease |  | - | Unknown |
| CHEMBL4070 | Casein kinase II alpha | Protein kinase |  | Pf3D7_1108400 | Protein phosphorylation |
| CHEMBL2096675 | Integrin α-V/β5 | Membrane receptor |  | Pf3D7_1475400 | Ligand/receptor binding |
| CHEMBL3024 | Serine/threonine protein kinase PLK1 | Protein kinase |  | Pf3D7_1246900 | Mitotic spindle assembly |
| CHEMBL4161 | Urease | Hydrolase |  | - | Unknown |
| CHEMBL4777 | Neuropeptide Y receptor type 1 | Membrane G Protein Coupled Receptor |  | - | Unknown |
| CHEMBL276 | Muscarinic acetylcholine receptor M1 | Membrane G Protein Coupled Receptor |  | Pf3D7_0100100 | Cytoadherence |
| CHEMBL5023 | p53-binding protein mdm-2 | Nuclear protein |  | - | Unknown |
| CHEMBL2411 | Serotonin 3a (5HT3a) receptor | Family A G protein-coupled receptor (ion channel) |  | Pf3D7_0710000 | Unknown |
| CHEMBL4123 | Neurotensin receptor 1 | Membrane Family A G protein coupled receptor |  | Pf3D7_1245200 | Transmembrane transport |
| CHEMBL326 | Alpha-1d adrenergic receptor | Membrane Family A G protein coupled receptor |  | Pf3D7_0207600 | Proteolysis |
| CHEMBL5451 | Sodium channel protein type X alpha subunit | Ligand-gated ion channel |  | Pf3D7_1433400 | Transmembrane protein of unknown function |
| CHEMBL4766 | Pyruvate dehydrogenase kinase isoform 1 | Protein kinase |  | Pf3D7_0517900 | Unknown |
| CHEMBL5568 | Proto-oncogene tyrosine protein kinase ROS | Protein kinase |  | Pf3D7_0211700 | Protein phosphorylation |
| CHEMBL1671613 | Type 1 angiotensin II receptor | Membrane Family A G protein coupled receptor |  | Pf3D7_1235200 | Ion homeostasis |
| CHEMBL5285 | Mitogen activated protein kinase kinase 5 | Protein kinase |  | Pf3D7_0610600 | Protein phosphorylation |
| CHEMBL4398 | Purinergic receptor P2Y2 | Membrane Family A G protein coupled receptor |  | Pf3D7_0812900 | Unknown |
| CHEMBL3137262 | LSD1/CoREST complex | Epigenetic regulator |  | Pf3D7_1211600 | Histone demethylation |
| CHEMBL3975 | Fructose-1,6-bisphosphatase | Enzyme |  | Pf3D7_1444800 | Glycolysis |
| CHEMBL5113 | Orexin receptor 1 | Membrane Family A G protein coupled receptor |  | - | Unknown |
| CHEMBL4608 | Melanocortin receptor 5 | Membrane Family A G protein coupled receptor |  | Pf3D7_1423400 | Conserved Plasmodium membrane protein, unknown function |
| CHEMBL5747 | CREB binding protein | Epigenetic regulator |  | Pf3D7_1212900 | Histone modification |
| CHEMBL1795139 | Transmembrane protease serine 6 | Serine protease |  | Pf3D7_1362900 | Unknown |
| CHEMBL2781 | Sodium/hydrogen exchanger 1 | Ligand-gated ion channel |  | Pf3D7_1303500 | Cytosolic pH regulation and quinine resistance |
| CHEMBL5141 | CYP26A1 | Cytochrome P450 |  | Pf3D7_1441900 | DNA damage repair |
| CHEMBL1947 | Thyroid hormone receptor beta 1 | Nuclear receptor |  | Pf3D7_1123100 | Cyclic nucleotide signalling |
| CHEMBL3774295 | Lysine-specific demethylase 5B | Epigenetic regulator |  | Pf3D7_0809900 | Histone modification |
| CHEMBL4791 | Endothelin converting enzyme 1 | Metalloprotease |  | Pf3D7_1010200 | DNA unwinding |
| CHEMBL5845 | Glycine receptor subunit 1α | Ligand-gated ion channel |  | Pf3D7_1405700 | A hypothetical membrane protein |
| CHEMBL235 | Perixosome proliferation-activated receptor gamma | Type II nuclear receptor |  | Pf3D7_1359300 | RNA processing |
| CHEMBL1287623 | Lethal (3) malignant brain tumor like protein 3 | Epigenetic transcription factor |  | Pf3D7_0904900 | Cu^2+^ ion transport |
| CHEMBL3816 | Cytosolic phospholipase A2 | Enzyme |  | Pf3D7_1038800 | RBC surface remodelling |
| CHEMBL3976 | Dipeptidyl peptidase II | Serine protease |  | Pf3D7_1247800 | Proteolysis |
| CHEMBL3037 | Cannabinoid CB1 receptor | Membrane Family A G protein coupled receptor |  | Pf3D7_1302500 | Unknown |
| CHEMBL1907603 | Glutamate NMDA receptor; GRIN1/GRIN2B | Ligand-gated ion channel |  | Pf3D7_0710200 | Unknown |
| CHEMBL2363 | Dihydrofolate reductase | Oxidoreductase |  | Pf3D7_0417200 | Folate metabolism |
| CHEMBL1849 | Enoyl-[acyl carrier protein] reductase | Oxidoreductase |  | Pf3D7_0615100 | Lipid metabolism |
| CHEMBL2096912 | Protein farnesyltransferase | Transferase |  | Pf3D7_1242600 | Protein farnesylation |
| CHEMBL1908385 | Serine/threonine protein kinase pknB | Protein kinase |  | Pf3D7_0610600 | Male gamete exflagellation |
| CHEMBL288 | Phosphodiesterase 4D | Phosphodiesterase |  | Pf3D7_1321500 | Signal transduction |
| CHEMBL1860 | Thyroid hormone receptor alpha | Nuclear receptor |  | Pf3D7_1123100 | Cyclic nucleotide signalling |
| CHEMBL1940 | Voltage-gated L-type calcium channel alpha 1C subunit | Ligand-gated ion channel |  | Pf3D7_0830500 | Putative amino acid transporter |
| CHEMBL2016 | Coagulation factor IX | Serine protease |  | Pf3D7_0323400 | Cytoadherence |
| CHEMBL3105 | Poly (ADP-ribose) polymerase 1 | Transferase |  | Pf3D7_0923100 | Ubiquitination reactions |
| CHEMBL3667 | PI4-kinase α-subunit | Transferase |  | Pf3D7_0509800 | Inositol Phosphate metabolism |
| CHEMBL1856 | Steroid 5-alpha reductase 2 | Oxidoreductase |  | Pf3D7_ 1135900 | Glycerophospholipid synthesis |
| CHEMBL1741186 | Nuclear receptor ROR-gamma | Nuclear receptor |  | - | Unknown |
| CHEMBL3891 | Calpain 1 | Cysteine protease |  | Pf3D7_1362400 | Nucleolar protein trafficking |
| CHEMBL3359 | Formyl peptide receptor 1 | Membrane Family A G protein coupled receptor |  | - | Unknown |
| CHEMBL5983 | Aldo-keto reductase family 1 membrane B10 | Oxidoreductase |  | Pf3D7_1364600 | Glyoxalase metabolism |
| CHEMBL1892 | Glutamate carboxypeptidase II | Metalloprotease |  | Pf3D7_1200600 | RBC invasion |
| CHEMBL3199 | Acetylcholinesterase | Serine hydrolase |  | - | Unknown |
| CHEMBL4051 | Cystic fibrosis transmembrane conductance regulator | Ion channel receptor |  | Pf3D7_1352100 | Transmembrane transport |

^a^Predictions performed at 70-90% confidence and only targets labelled “Active” selected.

^b^Malarial homology search based on *BLASTp* of target sequences in PlasmoDB. (-) denotes lack of homology in *Plasmodium* annotations.

**Table S5: Molecular docking of *Plasmodium* targets to isoliensinine**

| **Target** | **PlasmoDB ID** | **Swiss-Model template** | **Sequence identity (%)** | **QMEAN** | **GMQE** | **Ramachandran favoured (%)** | **Binding Affinity (kcal/mol)** |
| --- | --- | --- | --- | --- | --- | --- | --- |
| **Nek2** | Pf3d7_0525900 | 4apc.1.A | 38.02 | -1.79 | 0.64 | 95.11 | -10.8 |
| **CLK1** | Pf3d7_1445400 | 3llt.1.A | 100 | -0.79 | 0.21 | 97.05 | -10 |
| **Map2** | Pf3d7_1113900 | 3nie.1.A | 100 | -1.74 | 0.61 | 94.29 | -10 |
| **CLK4** | Pf3d7_0302100 | 6fad.1.A | 46.41 | -3.2 | 0.04 | 90.82 | -9.8 |
|  |  |  |  |  |  |  |  |
| **eEF2** | Pf3d7_1451100 | 1u2r.1 | 61.3 | -1.2 | 0.79 | 95.41 | -9.6 |
| **Nek1** | Pf3d7_1228300 | 5m57.1.A | 42.11 | -3.29 | 0.1 | 88.46 | -9.5 |
| **Sir2a** | Pf3d7_1328800 | 3jwp.1.A | 100 | -0.2 | 0.9 | 96.84 | -9.5 |
| **FNT** | Pf3d7_0316600 | 6vqq.1.D | 100 | -2.81 | 0.83 | 97.55 | -9.1 |
| **PKG** | Pf3d7_1436600 | 5dyk.1.A | 100 | -0.03 | 0.83 | 96.54 | -9.1 |
| **TKL1** | Pf3d7_0211700 | 6nsq.1.A | 28.3 | -3.53 | 0.05 | 92.04 | -9.1 |
| **CDPK4** | Pf3d7_0717500 | 4rgj.1.A | 100 | 0.23 | 0.78 | 97.95 | -9 |
| **PrP16** | Pf3d7_1364300 | 5yzg.1.0 | 50.12 | -4.31 | 0.4 | 85.7 | -9 |
| **CK2** | Pf3d7_1108400 | 5xvu.1.A | 99.69 | -0.57 | 0.87 | 95.92 | -8.9 |
| **CAX** | Pf3d7_0603500 | 4k1c.1.A | 44.73 | -4.41 | 0.57 | 97.13 | -8.9 |
| **Nek4** | Pf3d7_0719200 | 4apc.1.A | 40.34 | -2.31 | 0.63 | 94.58 | -8.9 |
| **CK1** | Pf3d7_1136500 | 6gzd.1.A | 62.73 | -1.37 | 0.79 | 96.23 | -8.9 |
| **Ark2** | Pf3d7_0309200 | 6gr9.1.A | 42.08 | -1.82 | 0 | 92.65 | -8.8 |
| **CRT** | Pf3d7_0709000 | 6ukj.1 | 98.82 | -4.25 | 0.74 | 96.08 | -8.7 |
| **Ark1** | Pf3d7_0605300 | 6npz.2.A | 25.32 | -4.36 | 0.55 | 93.08 | -8.6 |
| **Zipco** | Pf3d7_1022300 | 6pgi.1.A | 14.52 | -7.43 | 0.51 | 88.39 | -8.6 |
| **AP2-G** | Pf3d7_1222600 | 6sy0.1.A | 33.93 | -2.09 | 0 | 96.88 | -8.5 |
| **PKB** | Pf3d7_1246900 | 3iw4.1 | 40 | -3.34 | 0.22 | 91.69 | -8.5 |
| **Purine nucleoside phosphorylase** | Pf3d7_0513300 | 5znc.1.A | 100 | 0.22 | 0.94 | 96.67 | -8.5 |
| **PRMT5** | Pf3d7_1361000 | 4gqb.1.A | 33.06 | -3.71 | 0.6 | 86.11 | -8.5 |
| **MORN1** | Pf3d7_1031200 | 6t4d.1.A | 100 | 4.03 | 0.37 | 98.59 | -8.3 |
| **NPL domain-containing protein** | Pf3d7_0813300 | 6j2z.1.C | 25.77 | -3.61 | 0.11 | 88.48 | -8.2 |
| **MCM4** | Pf3d7_1317100 | 6eyc.1.C | 38.66 | -5.5 | 0.38 | 84.31 | -8.2 |
| **CDC20** | Pf3d7_1026400 | 6tlj.1.R | 40.76 | -5.33 | 0.31 | 87.78 | -8.2 |
| **GCN5** | Pf3d7_0823300 | 6cw3.1.C | 48.72 | -3.31 | 0.05 | 91.04 | -8.1 |
| **crk4** | Pf3d7_0317200 | 4rt7.1.A | 17.11 | -8.56 | 0.03 | 81.92 | -8 |
| **CDPK5** | Pf3d7_1337800 | 3q5i.1.A | 40.49 | -1 | 0.54 | 95.11 | -7.9 |
| **CN3** | Pf3d7_1027700 | 3kf9.1.A | 61.9 | 0.87 | 0.6 | 95.86 | -7.7 |
| **PK2** | Pf3d7_1238900 | 6w4o.1.M | 32.26 | -3.96 | 0.29 | 92.78 | -7.7 |
|  |  |  |  |  |  |  |  |
| **Abcg2** | Pf3d7_1426500 | 6vxf.1.B | 26.69 | -5.85 | 0.54 | 84.77 | -7.6 |
| **ATP6** | Pf3d7_0106300 | 3fgo.1 | 49.48 | -5.36 | 0.54 | 84.52 | -7.6 |
| **HMGB3** | Pf3d7_1205800 | 5n9g.1.C | 23.53 | -1.54 | 0 | 97.75 | -7.6 |
| **Myb1** | Pf3d7_1315800 | 7b9v.1.R | 16.42 | -1.58 | 0.13 | 88.32 | -7.6 |
| **CDPK7** | Pf3d7_1123100 | 2w4o.1.A | 39.93 | -3.75 | 0 | 90.58 | -7.6 |
| **CLK3** | Pf3d7_1114700 | 6qx9.56.A | 53.76 | -2.58 | 0.31 | 93.43 | -7.5 |
| **DHPS** | Pf3d7_0810800 | 6jwy.1.A | 100 | -3.89 | 0.74 | 89.35 | -7.5 |
| **ORC3** | Pf3d7_1029900 | 4xgc.1.B | 18.84 | -3.02 | 0 | 92.03 | -7.5 |
| **MDR1** | Pf3d7_0523000 | 6ujp.1 | 31.48 | -5.08 | 0.49 | 88.44 | -7.3 |
| **Dhfr-TS** | Pf3d7_0417200 | 3qgt.1.A | 100 | -1.99 | 0.87 | 92.33 | -7.2 |
| **HMGB1** | Pf3d7_1202900 | 2lhj.1.A | 64.77 | -2.78 | 0.64 | 95.35 | -7.2 |
| **SUB1** | Pf3d7_0507500 | 4tr2.1.A | 60.7 | -2.97 | 0.64 | 88.71 | -7.2 |
| **LSD1** | Pf3d7_1211600 | 3zmu.1.A | 25.48 | -4.48 | 0 | 89.86 | -7.1 |
| **MDR6** | Pf3d7_1339900 | 4myc.1.B | 35 | -5.38 | 0.23 | 85.7 | -7.1 |
| **SRSF12** | Pf3d7_0503300 | 7csx.1.A | 27.13 | -1.5 | 0.15 | 96.09 | -7 |
| **DIS3** | pf3d7_1359300 | 6h25.1.J | 32.04 | -5.15 | 0.56 | 88.45 | -7 |
| **GDV1** | Pf3d7_0935400 | 2qip.1.A | 15.83 | -3.3 | 0.07 | 87.59 | -6.9 |
| **APC3** | Pf3d7_0501700 | 5a31.1.F | 17.32 | -2.97 | 0.15 | 95.72 | -6.9 |
| **SFRS4** | Pf3d7_1022400 | 5ifm.5.A | 26.62 | -2.73 | 0.12 | 90.91 | -6.9 |
| **GSK3** | Pf3d7_0312400 | 1q3d.1.B | 54.65 | -2.1 | 0.57 | 92.97 | -6.8 |
| **NCR1** | Pf3d7_0107500 | 6v3h.1.A | 22.28 | -8.33 | 0.28 | 80.34 | -6.7 |
| **NF-YB** | Pf3d7_1146600 | 1jfi.1.D | 30.97 | -0.33 | 0 | 97.3 | -6.6 |
| **SAP18** | Pf3d7_0711400 | 4a9o.1.A | 36.84 | -2.76 | 0.06 | 89.47 | -6.6 |
| **SF1** | Pf3d7_1321700 | 6r5k.1.E | 25 | -6.58 | 0.15 | 83.24 | -6.5 |
| **ORC5** | Pf3d7_0215800 | 7jgs.1.A | 23.33 | -7.41 | 0.27 | 79.07 | -6.4 |
| **Pkar** | Pf3d7_1223100 | 5kbf.1.A | 100 | -0.2 | 0.49 | 96.56 | -6.4 |
| **Copper Atpase** | Pf3d7_0904900 | 4bbj.1.A | 24.43 | -9.05 | 0 | 78.55 | -6.3 |
| **HMGB2** | Pf3d7_0817900 | 2mrc.1.A | 100 | 0.15 | 0.69 | 96.3 | -6.3 |
| **SMD1** | Pf3d7_1125500 | 5zwn.1.k | 44.9 | -3.35 | 0.48 | 90.62 | -6.2 |
| **Calpain** | Pf3d7_1362400 | 6bjd.4.A | 24.25 | -9.18 | 0.03 | 81.41 | -6.2 |
| **CDPK1** | Pf3d7_0217500 | 3q5i.1.A | 94.42 | -0.55 | 0.79 | 95.84 | -6.1 |
| **NOP5** | Pf3d7_1008800 | 6zqd.27.A | 45.01 | -3.32 | 0.66 | 91.22 | -6 |
| **PK5** | Pf3d7_1356900 | 1v0p.1.A | 99.65 | -0.16 | 0.89 | 95.77 | -6 |
| **BDP2** | Pf3d7_1212900 | 1e6i.1.A | 32.41 | -1.2 | 0.03 | 98.13 | -5.7 |
| **SR1** | Pf3d7_0517300 | 7abg.1.f | 52 | -2.49 | 0.15 | 83.24 | -5.7 |
| **PI4K** | Pf3d7_0509800 | 4wae.1.A | 43.94 | -2.06 | 0.06 | 94.21 | -4.9 |
| **NDC80** | Pf3d7_0616200 | 6ian.1.B | 12.96 | -4.25 | 0.26 | 88.94 | -4.5 |
| **ATP4** | Pf3d7_1211900 | 6jxh.1 | 29.52 | -4.89 | 0.44 | 89.27 | -4.4 |
| **HDAC2** | Pf3d7_1008000 | 1zz0.1.A | 38.84 | -7.09 | 0 | 76.75 | -4.2 |
| **CYC1** | Pf3d7_0518400 | 7egb.1.J | 19.19 | -4.49 | 0.49 | 90.69 | -2.9 |
| **HDAC1** | Pf3d7_1472200 | 1zz0.1.A | 26.92 | -9.09 | 0 | 79.59 | -0.4 |
| **DHODH** | Pf3d7_0603300 | 6vty.1.A | 100 | -0.61 | 0.54 | 95.82 | 0 |
| **CLK2** | Pf3d7_1443000 | 4ifc.1.A | 25.25 | -8.85 | 0.07 | 75.94 | 1.8 |
| **JmJC1** | Pf3d7_0809900 | 5ykn.1.A | 29.56 | -8.05 | 0.23 | 78.51 | 2.8 |
| **CPSF3** | Pf3d7_1438500 | 6v4x.1.H | 39.74 | -4.65 | 0.41 | 84.75 | 3.2 |
| **KRS1** | Pf3d7_1350100 | 6hcu.1.B | 100 | -0.36 | 0.75 | 94.93 | 4.9 |
| **HT1** | Pf3d7_0204700 | 6m20.1.A | 100 | -2.3 | 0.84 | 98.31 | 5.4 |
| **Tubulin 1** | Pf3d7_0903700 | 6dpv.1.E | 83.96 | -1.73 | 0.84 | 95.63 | 9.8 |
| **Tubulin 2** | Pf3d7_0422300 | 6dpv.1.E | 83.33 | -1.65 | 0.84 | 95.86 | 10.9 |
| **AQP** | Pf3d7_0810400 | 3llq.1 | 22.6 | -10.9 | 0.11 | 78.77 | 20.5 |
| **VP2** | Pf3d7_1235200 | 6afx | 37.77 | -5.41 | 0.45 | 91.15 | 28.3 |

**Table S6: ADME prediction profile enlisting of isoliensinine from SWISSADME platform.**

| **Property** | **Values** |
| --- | --- |
| **Molecular weight (MW)** | 610.74 |
| **Consensus lipophilicity (Log P_o/w_)** | 5.16 |
| **Water solubility** | Poor (LogS = -7.45) |
| **TPSA** | 83.86 |
| **BBB permeant** | No |
| **P-gp inhibitor** | Yes |
| **P-gp substrate** | Yes |
| **CYP1A2 inhibitor** | No |
| **CYP2C19 inhibitor** | No |
| **CYP3A4 inhibitor** | No |
| **CYP2C9 inhibitor** | No |
| **CYP2D6 inhibitor** | No |
| **GI absorption** | High |
| **Bioavailability score** | 0.55 |
| **Lipinski drug-likeness** | Yes; 1 violation: MW>500 |
| **PAINS** | No |
